# Is fever good? What do we actually know?

**DOI:** 10.1186/1471-227X-15-S1-A17

**Published:** 2015-06-24

**Authors:** Jonathan KJ Rhodes

**Affiliations:** 1 Department of Anaesthesia, Critical Care and Pain Management, University of Edinburgh, UK

## 

In patients with acquired brain injury there is a widely held belief that fever is harmful. However, a critical examination of the literature suggests that these beliefs may be based more on faith and less on science.

Cooling the injured brain to prevent further injury makes intuitive sense and is supported by a diverse array of animal studies. However, to date, data from good quality randomised controlled trails proving that therapeutic hypothermia is beneficial are lacking. Indeed the recent Targeted Temperature Management Trial found no additional advantage to cooling below normothermia following cardiac arrest [1]. However, this result would support the argument that the benefit of targeted temperature management is due to the avoidance of fever.

On the face of it this hypothesis is supported by experimental studies. However, these tend to employ induced hyperthermia which is quite different from spontaneous endogenous fever.

In clinical studies fever is widely reported as associated with adverse outcomes following cardiac arrest, brain trauma and stroke. However, proving the causality is problematic as this could merely be an association with injury severity through the induction of inflammatory systems [2]. In an attempt to control for such confounding, the technique of multivariable logistic regression is commonly used to suggest that fever is an independent variable associated with poor outcome [3]. However, the prediction of outcome variability depends on the robustness of the model used and so it is always possible that fever captures some aspect of severity that is not captured by other variables.

Whilst there is a clear association between fever and poor outcome following acute brain injury, it should be remembered that that causality remains unproven. Furthermore, and most significantly, there are no trials demonstrating that preventing or treating fever following brain injury improves outcome. Clearly this is an area in need of more scientific study.

## Declaration

The author is supported by an award from National Health Research Scotland. JKJR has received honorariums from BARD for lecturing. BARD had no input into the topics covered in this abstract.
